# The Drosophila Cadherin Fat Regulates Tissue Size and Planar Cell Polarity through Different Domains

**DOI:** 10.1371/journal.pone.0062998

**Published:** 2013-05-07

**Authors:** Xuesong Zhao, Chung-hui Yang, Michael A. Simon

**Affiliations:** Department of Biology, Stanford University, Stanford, California, United States of America; University of Dayton, United States of America

## Abstract

The *Drosophila* Cadherin Fat (Ft) has been identified as a crucial regulator of tissue size and Planar Cell Polarity (PCP). However, the precise mechanism by which Ft regulates these processes remains unclear. In order to advance our understanding of the action of Ft, we have sought to identify the crucial Ft effector domains. Here we report that a small region of the Ft cytoplasmic domain (H2 region) is both necessary and sufficient, when membrane localized, to support viability and prevent tissue overgrowth. Interestingly, the H2 region is dispensable for regulating PCP signaling, whereas the mutant Ft lacking the H2 region is fully capable of directing PCP. This result suggests that Ft’s roles in PCP signaling and tissue size control are separable, and each can be carried out independently. Surprisingly, the crucial regions of Ft identified in our structure-function study do not overlap with the previously reported interaction regions with Atrophin, Dco, or Lowfat.

## Introduction

The *Drosophila* Fat (Ft), a large 34 cadherin domain-containing transmembrane adhesion molecule, plays a key role in the establishment of epithelial planar cell polarity (PCP) [1], [2], [3]. PCP is a form of polarity along an axis within the plane of an epithelium. It is manifested in many *Drosophila* tissues, including the uniform orientation of hairs and bristles on the adult body and wing, and the precise alignment of ommatidia in the eye. The role of Ft has been proposed to provide a directional signal for aligning PCP [1], [2]. It has been intensively investigated how Ft and another group of conserved signaling molecules, core PCP proteins, are working together to mediate the PCP signaling [4], [5], [6], [7], [8], [9]. Although considerable insight has been gained into the core PCP pathway, how Ft tranduces downstream PCP signal remains unclear.

In addition to regulating PCP, Ft has also been identified as a negative regulator of tissue growth. Animals lacking Ft function exhibit massive overgrowth of all imaginal discs, the larval epithelial tissues which ultimately form much of the adult body. Mutant animals subsequently die as pupae [10], [11]. In addition to the overall increase in tissue size seen in animals lacking Ft function, studies of *ft* mutant cells in mosaic animals have shown that cells lacking Ft function proliferate much more extensively than their wild-type neighbors [12]. Each of these observations suggests that cells lacking Ft are unable to respond to signals that normally limit their growth and proliferation.

Significant advancements have been made to elucidate the downstream pathway of Ft in past few years [13], [14], [15], [16]. Several genetic studies have demonstrated that the Hpo/Wts signaling pathway is an important target of Ft in tissue growth regulation [17], [18], [19], [20], [21]. The core of the Hpo/Wts signal transduction system is a protein kinase cascade consisting of the serine/threonine kinases Hippo (Hpo) [22], [23], [24], [25], [26] and Warts (Wts) [27], [28], and their associated scaffolding proteins Salvador (Sav) [29], [30] and Mats (Mob as tumor suppressor) [31]. Upon activation of the kinases, the transcriptional activator Yorkie (Yki) is phosphorylated and inactivated, thus leading to reduced expression of Yki targets [32], [33], [34]. These targets include proliferation promoting factors such as Cyclin E and anti-apoptotic factors such as DIAP1 [25], [30]. As a result, activation of the Hpo/Wts pathway leads to reduced cellular proliferation and increased cell death [35], [36], [37]. In contrast, inactivation of the Hpo/Wts protein kinase cascade in clones leads to elevated expression of Yki targets and overproliferation in a manner similar to clones of cells lacking Ft function. The similarity of clonal phenotypes as well as the induction of Yki targets in *ft* mutant cells indicates that Ft regulates Hpo/Wts signaling. Genetic studies also placed Dachs, an unconventional myosin, Approximated, a palmitoyltransferase, and Expanded, a tumor suppressor, between Ft and downstream Hpo/Wts regulation and/or PCP signaling [17], [18], [20], [38], [39]. However, due to lack of biochemical link and complex nature of Ft signaling, the precise mode of action of Ft is not understood.

Several binding partners of Ft cytoplasmic domain have been identified and proposed to carry out some of Ft downstream activities. Atrophin/Grunge, a transcriptional repressor, which binds to the C-terminal fragment of Ft, has been proposed to mediate downstream PCP signaling [40]. A conserved cytoplamic protein, Lowfat and its human homologs LIX1 and LIX1-like, bind to the cytoplasmic domain of Ft and modulate Ft signaling by influencing the sub-apical membrane localization of Ft [41]. The Casein Kinase Discs overgrown (Dco) can bind to and phosphorylate the cytoplasmic domain of Ft, and control Ft signaling in both growth and PCP [42], [43]. However, it has not been examined whether these interaction regions in Ft are crucial for regulating in PCP and tissue growth *in vivo*.

In order to further investigate the downstream signaling pathways regulated by Ft, we sought to identify regions of Ft that are essential for Ft function. Here, we identify a crucial region (H2 region) in the Ft cytoplasmic domain that, when membrane localized, is both necessary and sufficient to support viability and limit tissue size in animals lacking wild-type Ft function. Interestingly, this region is dispensable for PCP, indicating that Ft uses different regions to regulate PCP and tissue size. Moreover, the crucial regions of Ft identified in our structure-function study do not overlap with the previously reported interaction regions with Atrophin, Dco, or Lowfat.

While we are preparing for the manuscript, a similar structure-function study was published based on analysis in the wing [44]. In comparison, the conclusion from this report is largely in agreement with our results with a few exceptions. Both studies have mapped to the same region in the cytoplasmic domain of Ft that is required to support viability and tissue size regulation, but dispensable for PCP. Surprisingly, in our observation, expression of the H2 region does not have discernible ability to activate Hpo/Wts signaling in the eye. In addition, the extracellular domain of Ft is essential for PCP signaling in the eye, but dispensable in the wing, consistent with previous observations of tissue-specific difference for regulating Ft PCP signaling [3], [45]. Together, our study in the eye and the recent report in the wing support the idea that Ft regulates PCP and tissue growth through separated functional regions.

## Materials and Methods

### Fly Stocks

The following stocks were used: *ft^G−rv^* [10] and *ft^l(2)fd^* [11], *E(spl)mδ0.5* (R4 cell fate marker) [46], *fj-lacZ* [47]. The Gal4 drivers used were TubP-Gal4 [48], Omb3-Gal4 and Vg1-Gal4 [49], and Flip-out Gal4 (ActP>STOP, y+ >Gal4) [50]. FLP/FRT [51] was used to generate mutant clones in imaginal discs. All rescue experiments were performed in *ft* null mutant combination *ft^G−rv^*/*ft^l(2)fd^*. UAS-FtΔECD [52].

### Expression Constructs

The full-length Ft (Ft-FL) construct was generated as previously described [3]. Ft-FL, FtΔcyt, FL-ΔH2, FL-ΔH345 were placed into the pUASP vector, while all the intracellular mutants were in the pUAST vector [53], [54]. Each mutant construct has a V5 tag at the C-terminus. The V5 tag does not interfere with function as full-length Ft with or without the V5 tag provides the same degree of rescue in lethality, disc size, DIAP1 expression and PCP assays (data not shown). Each intracellular domain mutant construct has a myristylation sequence at N-terminus. The myristylation sequence used was: MGNKCCSKRQ [55]. The mutated version (Myr-mut) of Ft-intra construct contains a (GA) point mutation in myristylation sequence, which inactivates the myristylation signal. FtΔECD is described in [52].

Amino Acids (a.a.) numbering is based on the protein sequence from Swiss-Prot: P33450. Sequences of Ft mutants are: Ft-FL: 1–5147a.a., FtΔcyt: 1–4624a.a., FL-ΔH2: full length with deletion of 4719–4900a.a., FL-ΔH345: full length with deletion of 4897–5147a.a., Ft-intra: 4612–5147a.a., MH1234∶4612–5016a.a., MH123∶4612–4966a.a., MH12∶4612–4900a.a., MH1∶4612–4750a.a., MH2∶4719–4900a.a., MH45∶4967–5147a.a., MH345∶4897–5147a.a., MH2345∶4719–5147a.a., MΔH2: intracellular domain sequence with deletion of 4719–4900a.a. Complete sequences of those constructs are available on request.

### Sections and Immunohistochemistry

Tangential sectioning of adult eyes was performed as previously described, except that the osmium steps were omitted [56]. Third instar eye discs were dissected in cold PBS and fixed in 4% formaldehyde for 45 min on ice. Antibodies used were as follows: Mouse anti-DIAP1 (1∶200; a gift from B. Hay), Rat anti-CycE (1∶400; a gift from H. Richardson), Mouse anti-β-gal(1∶1000; Promega), Rabbit anti-GFP (1∶1000; Molecular Probes), Chicken anti-GFP (1∶1000; Molecular Prob), and Rabbit anti-V5 (1∶5000, Sigma). Cy3, Cy5, and FITC-conjugated secondary antibodies (Jackson Lab) were used at 1∶200 dilution. Fluorescein phalloidin was used at 1∶100 dilution. Confocal pictures were taken with a Biorad MRC 1024 microscope.

### Immunoblotting

Third-instar larval imaginal discs were lysed and boiled in protein sample buffer. Samples were subjected to SDS-PAGE, transferred to Hybond-P membrane, and probed with Rabbit anti-V5 (1∶10,000; Sigma), Rabbit anti-DIAP1 (1∶5000; a gift from K. White) or Mouse anti-β-tubulin (1∶10,000; DSHB). Secondary antibodies used were Rabbit and Mouse anti-HRP.

For comparisons of DIAP1 protein levels, 20∼30 eye imaginal discs were collected for each indicated genotype for each immunoblot analysis. Using NIH ImageJ software, DIAP1 levels were quantified and normalized to β-tubulin, then normalized to the value in *wt* animals. Quantification result represents the mean of 3 independent experiments. Error bars show ± S.D. To assess statistical significance, data was analyzed by one-way ANOVA with post-hoc Bonferroni correction. Significance was placed at p<0.05.

## Results

### Membrane Localized Cytoplasmic Domain of Ft is Essential to Support Viability and Limit Tissue Size

**Figure 1:**
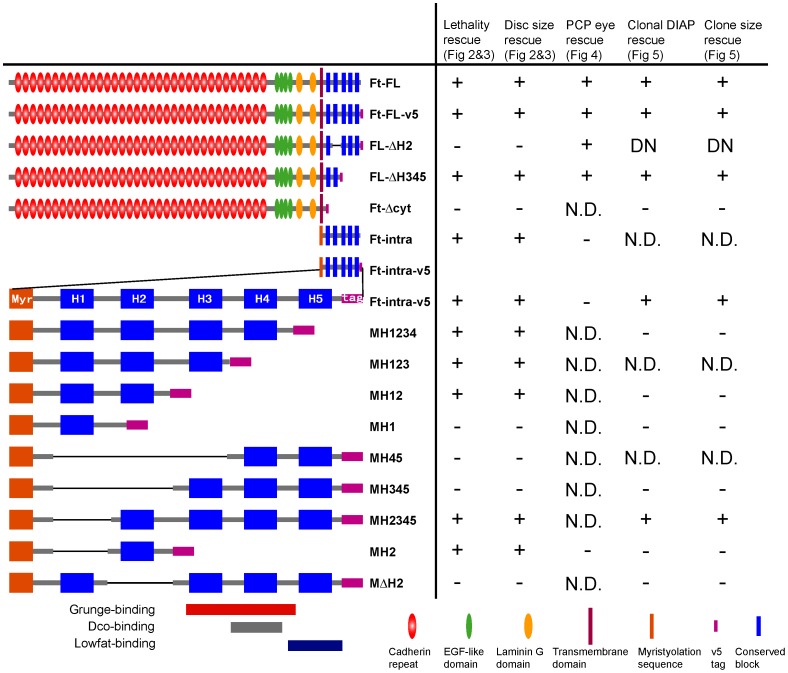
Summary of Ft constructs and their ability to rescue *ft* mutant phenotype. Schematic structure of Ft mutant constructs is on the left with their short names. (See Materials and Methods for details.) Summary of rescue results is on the right. Measurement and quantification of each rescue assay can be found in indicated figures. In the lethality rescue assay, effective rescue (+) was determined by at least 25% recovery of the expected number of *ft^G−rv^*/*ft^l(2)fd^; TubP-Gal4/UAS-transgene* adults, whereas no rescue (−) was determined for those transgenes which did not produce any adult animal (eclosion). In the eye disc size rescue assay, effective rescue (+) was determined by <20% increase of size compared to *wt* disc. In the PCP rescue assay, effective rescue (+) was determined by >85% correct orientation of ommatidia; (−) indicates disorganized polarity that the equator was unable to be recognized, and <75% correct orientation can be determined. In the clonal DIAP1 and clone size assays, effective rescue (+) was determined by >60% of clones with successful rescue of clonal phenotype and DIAP1 expression level. ‘DN’ indicates dominant negative effect of FL-ΔH2. N.D.: not determined.

**Figure 2:**
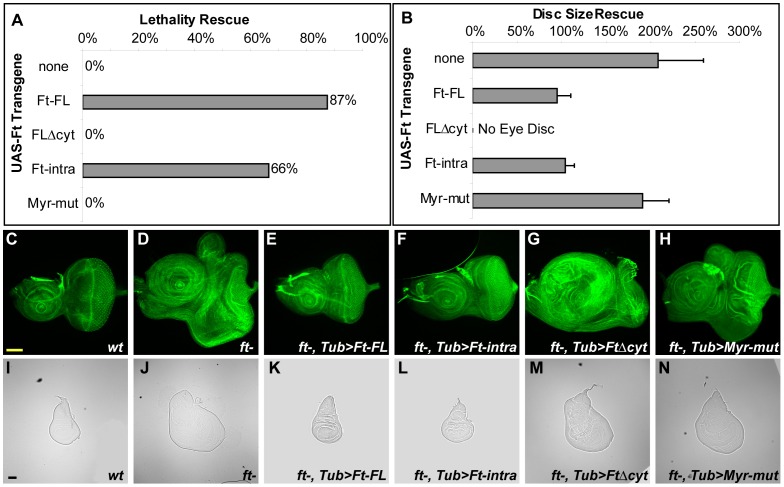
The intracellular domain of Ft is necessary and sufficient to rescue lethality and tissue size phenotype. (A) Quantification of the lethality rescue. Rescued adult (eclosed) flies were counted to compare with the expected number of *ft^G−rv^*/*ft^l(2)fd^; TubP-Gal4/UAS-transgene* adults. 100% indicates the expected number based on mendalian ratios. (B) Quantification of the eye disc size rescue. Third-instar eye discs from rescued animals were measured and compared to the size of the *wt* eye disc. 100% indicates the *wt* disc size. *ft* mutant disc is about twice the size of *wt* disc. (C–H) Third-instar eye imaginal discs stained with fluorescein phalloidin. Anterior is to the left and dorsal is up for all eye discs. Scale bar: 100 µM (I–N) Third-instar wing imaginal discs. Genotypes are: (C, I) *wt*, (D, J) *ft−/−*, (E, K) *ft−/−; TubP-Gal4/UAS-Ft-FL*, (F, L) *ft−/−; TubP-Gal4/UAS-Ft-intra*, (G, M) *ft−/−; TubP-Gal4/UAS-FtΔcyt,* (H, N) *ft−/−; TubP-Gal4/UAS-Myr-mut.*

In order to understand how Ft transduces growth regulatory signals to downstream pathways, we sought to identify regions of Ft that are essential for regulating tissue size. A series of deletion mutants of Ft were constructed, expressed *in vivo*, and assayed for their ability to rescue both the pupal lethality and eye imaginal disc overgrowth phenotypes associated with *ft* mutant animals (*ft^l(2)fd^*/*ft^G−rv^*). For this analysis, ubiquitous expression of the deleted Ft proteins was driven using the UAS/Gal4 system and a ubiquitous Tub-Gal4 driver. For each deletion mutant transgene, 2–3 independent insertions were analyzed. Schematic structures of Ft mutants and assay results are summarized in Figure 1. As expected, expression of the full-length wild-type Ft protein (Ft-FL) fully rescued pupal lethality (Figure 2A). Examination of late 3^rd^ instar eye discs from the rescued animals further showed that eye imaginal discs of rescued animals were normal in size compared to overgrown discs of mutant animals (Figure 2B&E).

We began our mutant analysis with the generation of Ft proteins in which either the entire intracellular domain was deleted and replaced by a V5 epitope tag (FtΔcyt) or in which the extracellular and transmembrane domains were replaced by a membrane targeting Src myristylation motif (Ft-intra) (Figure 1). Consistent with a previously published report [52], we found that ubiquitous expression of Ft-intra was sufficient to rescue both the lethality and disc size overgrowth phenotypes of *ft* mutant animals (Figure 1F). We further found that expression of either Ft-FL or Ft-intra under Eyeless-Gal4 control could restore eye imaginal disc size to normal, but not that of other tissues (Figure S1). This indicates that the ability of Ft to regulate tissue size is a tissue-autonomous function.

In contrast to the ability of Ft-FL and Ft-intra to regulate tissue size, expression of FtΔcyt did not support the viability of *ft* mutant animals. Examination of mutant larvae expressing FtΔcyt showed that eye discs appeared to have severe patterning defects and thus could not easily be evaluated for their overgrowth phenotype (Figure 2G). To assay the ability of FtΔcyt to regulate disc size, the size of wing discs from *ft* mutant animals expressing FtΔcyt was examined. While expression of the Ft-FL or Ft-intra in *ft* mutant animals restored their wing discs to normal size, the wing discs of *ft* mutant animals expressing FtΔcyt remained overgrown (Figures 2I–N). Taken together, these results indicate that the intracellular domain of Ft contains essential regions necessary for supporting viability and limiting tissue size.

The ability of membrane targeted Ft-intra to rescue the *ft* mutant phenotype raised the question of whether localization to the plasma membrane is required for Ft function in these rescue assays. To address this issue, a mutant form (Myr-mut) of Ft-intra was generated with a point mutation in the myristylation sequence that blocks myristylation and thus prevents membrane localization. This mutation abolishes Ft function in both the lethality and imaginal disc size rescue assays (Figures 2H, N), indicating that membrane localization is crucial for Ft function.

### A Small Region of the Intracellular Domain can Provide Ft Function for Regulating Viability and Tissue Size

**Figure 3:**
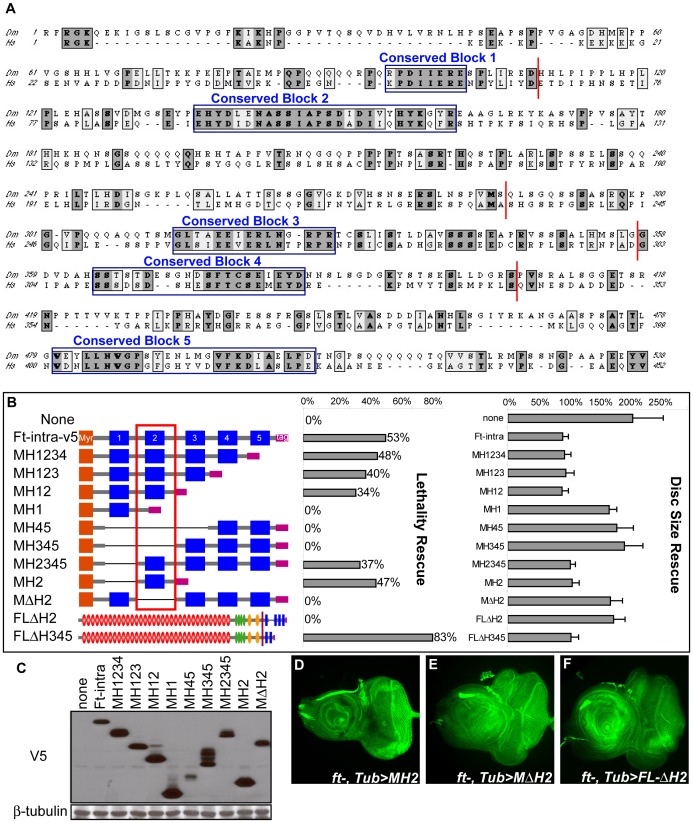
The H2 region is necessary and sufficient for viability and tissue size regulation. (A) Sequence alignment of cytoplasmic domain of Drosophila Ft (538 a.a.) and human Ft4 (452 a.a.) reveals highly conserved regions (conserved block 1–5) shown in blue boxes. The starting and ending positions of the deletion mutants are indicated by red bars. (B) Schematic structure of Ft intracellular domain deletion mutants is on the left. Myristylation sequence is indicated in orange. Conserved homology regions are indicated in blue. V5 tag is indicated in pink. Regions deleted are shown with black lines. Quantification of the lethality rescue and disc size rescue on the right. (See Fig. 2AB for method details) (C) Expression levels of intracellular domain deletion mutants are revealed by an immunoblot of imaginal disc extracts from *ft* mutant animals expressing various transgenes. Blots were probed with anti-V5 and anti-β-tubulin. (D–F) Deletion of the H2 region in either the intracellular domain (E) or the full-length Ft protein (F) effectively abolished rescuing ability, whereas the H2 region, when membrane localized (D), is necessary and sufficient to rescue both lethality and disc size overgrown phenotype. Third-instar eye imaginal discs were stained with fluorescein phalloidin. Genotypes are: (D) *ft−/−;TubP-Gal4/UAS-MH2,* (E) *ft−/−; TubP-Gal4/UAS-MΔH2,* (F) *ft−/−; TubP-Gal4/UAS-FL-ΔH2.*

To identify crucial regions within the intracellular domain of Ft, a series of deletion mutations that removed portions of Ft-intra were constructed and assayed for function. To guide this process, the cytoplasmic sequence of *Drosophila* Ft was compared to its homologs from other species. Sequence alignment reveals several highly conserved blocks of amino acids, referred to as conserved blocks 1–5 (Figure 3A). A series of mutant constructs were then generated in which the regions containing the conserved blocks were successively deleted from the N- or C-terminus of the Ft-intra protein (Figure 3B). Since membrane localization is required for growth control, each mutant protein was directed to the plasma membrane by a myristylation sequence at its N-terminus. In addition, the presence of a V5 epitope tag at the C-terminus of each protein allowed us to demonstrate that the various mutant proteins were expressed at similar levels (Figure 3C). Truncated proteins were then tested for their ability to rescue the lethality and disc overgrowth phenotype. The ability of the different truncated proteins to rescue *ft* mutant animals is summarized in Figures 3B, and examples in 3D–F.

This functional analysis led to the identification of a small region (H2) that is crucial for supporting viability and limiting tissue size (Figure 3B). Importantly, each of the truncated proteins lacking H2 was unable to rescue *ft* mutant phenotypes. In contrast, every protein containing the H2 region retained rescuing ability. The striking correlation between the presence of H2 and rescuing ability suggested that the H2 region might be both necessary and sufficient for effective Ft function. In order to test these possibilities directly, two additional mutant constructs were generated. In one, only the H2 region was deleted from Ft-intra (MΔH2). This protein failed to provide Ft function. In the second, only the H2 region of Ft was present in the expressed protein (MH2). This protein provided effective rescuing function. Taken together, these results indicate that the H2 region is both necessary and sufficient for Ft function in these assays. Consistent with the importance of the H2 region, its deletion from the full-length Ft protein (FL-ΔH2) effectively abolishes rescuing ability (Figure 3F).

### Regions Crucial for PCP Signaling in the Eye

**Figure 4:**
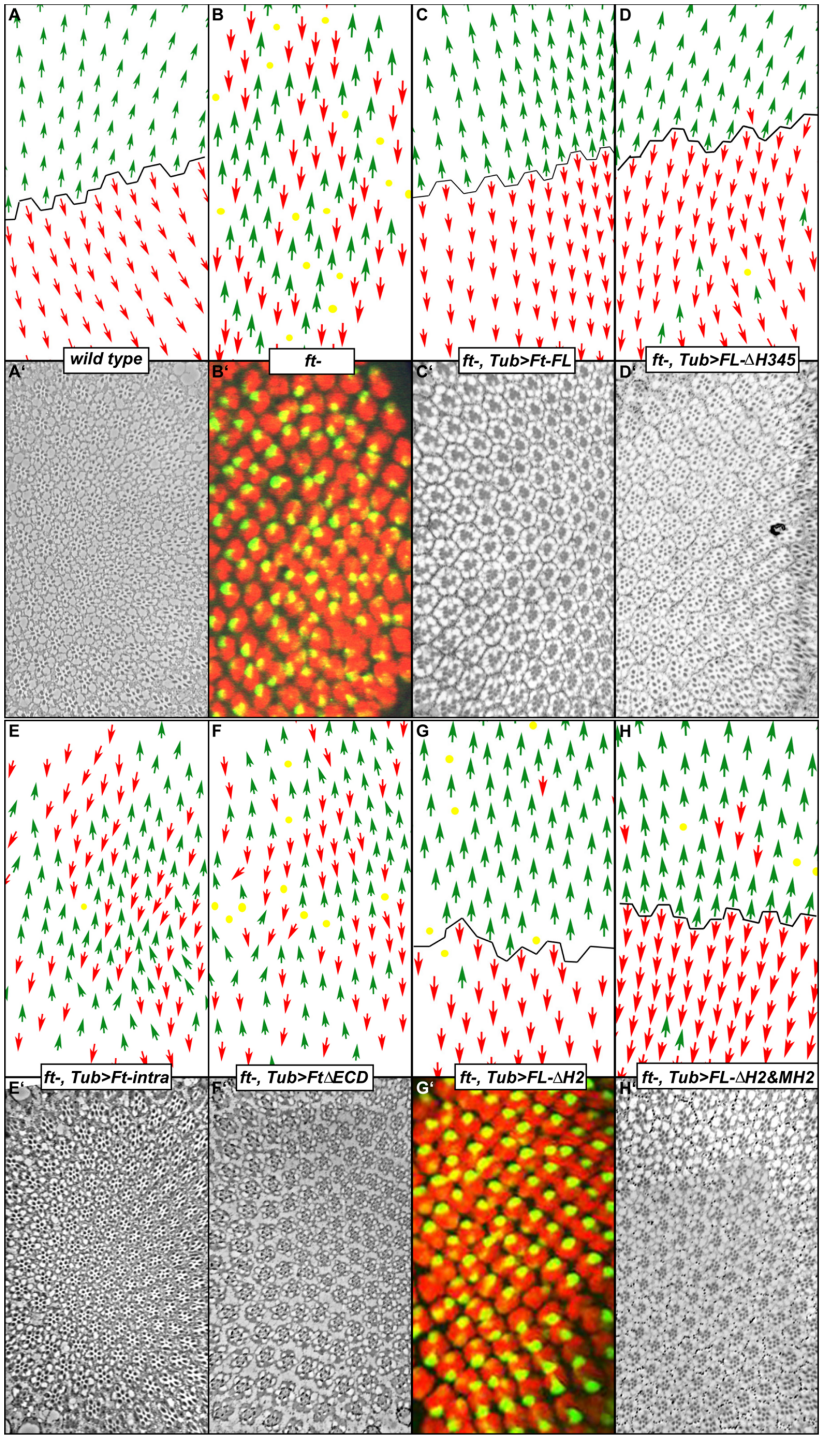
The H2 region is not required for Ft in regulating PCP signaling. Schematic diagrams (A–H) of equatorial sections of adult eyes (A’, C’, D’, E’, F’, H’), or confocal images of third instar eye imaginal discs (B’, G’). (B’, G’) Ommatidial orientation was determined by examining the expression of *E(spl)mδ0.5* (R4 marker) in each ommatidium. This marker consists of a portion of the enhancer region of the *E(spl)* gene, a transcriptional target of N activation, fused to a β-galactosidase reporter. This marker is strongly expressed in R4 cell (green) [46]. Elav staining (red) labels all photoreceptor cells in each ommatidial cluster. All images are oriented anterior to the left and dorsal up. The equator is indicated by the black line. Green arrows indicate ommatidia with dorsal chirality and orientation, whereas red arrows designate ventral type ommatidia. Yellow dots represent either incorrectly constructed ommatidia, ommatidia exhibiting severe misrotation or ommatidia with undetermined polarity. Relevant changes from wild type with regard to Ft function or expression are indicated at the bottom of each panel. Genotypes are listed below along with the fidelity rate (the percentage of ommatidia that are correctly constructed and rotated). Ubiquitous expression of Ft-FL, FL-ΔH2 or FL-ΔH345 can restore the wild-type pattern of ommatidia in *ft* mutant animals. FL-ΔH2 and FL-ΔH345 have a low rate of errors. (A, A’) *w^1118^* (100%) (B, B’) *w^1118^; ft−/−* (Disorganized, n = 430) (C, C’) *w^1118^; ft−/−; TubP-Gal4/UAS-Ft-FL* (99%, n = 788) (D, D’) *w^1118^; ft−/−; TubP-Gal4/UAS-FL-ΔH345* (91.1%, n = 529) (E, E’) *w^1118^; ft−/−; TubP-Gal4/UAS-Ft-intra* (Disorganized, n = 1592) (F, F’) *w^1118^; ft−/−; TubP-Gal4/UAS-FtΔECD* (Disorganized, n = 907) (G, G’) *w^1118^; ft−/−; TubP-Gal4/UAS-FL-ΔH2* (93.5%, n = 660) (H, H’) *w^1118^; ft−/−; TubP-Gal4/UAS-FL-ΔH2 & UAS-MH2* (91.5%, n = 471).

In addition to regulating viability and tissue size, Ft also plays a key role in the establishment of epithelial planar cell polarity (PCP) [1], [2], [3]. In the eye, planar cell polarity is apparent in the orientation of the ommatidial units that comprise the compound eye. In wild type, all of the ommatidia in the dorsal half of the eye are oriented in the same direction while those in the ventral region uniformly adopt the opposite chiral form (Figure 4A, A’). The establishment of this ordered pattern of polarity is dependent on Ft, whose absence leads to a randomized pattern of dorsal and ventral type ommatidia throughout the eye (Figure 4B, B’).

The importance of the intracellular domain (Ft-intra) for regulating viability and tissue size naturally raised a question as to whether Ft-intra is also necessary and sufficient for planar cell polarity signaling. To test this hypothesis, we carried out an eye PCP rescue experiment with Ft-intra. We have previously shown that ubiquitous Tub-Gal4 driven expression of Ft can fully re-establish the wild-type pattern of ommatidia in *ft* mutant animals (error rate of <1%) [3](Figure 4C, C’). This assay allowed us to examine whether Ft-intra could provide PCP function in *ft* mutant animals.

We found that uniform expression of Ft-intra cannot restore the overall pattern of polarization (Figure 4E, E’). A different version of the intracellular domain (FtΔECD) [52], which has the endogenous transmembrane and cytoplasmic domains, also fails to rescue the pattern of polarization (Figure 4F, F’). These findings indicate that in the eye, the Ft intracellular domain alone is not sufficient to provide all the function of Ft in PCP, suggesting that the extracellular domain is essential for receiving and/or directing the downstream PCP signaling. This is in marked contrast to the observation in the wing, where the Ft transmembrane and cytoplasmic domains together are sufficient to mediate PCP signaling [44], [52].

### Ft’s Role in PCP is Independent from Viability and Tissue Size Regulation

Although Ft-intra is not sufficient to rescue the PCP defect, it may still contain key regions necessary in PCP signaling. This is particularly interesting for the H2 region, given its key role for rescuing viability and tissue size. To test this hypothesis, and to extend it to the other regions of Ft, we tested two full length mutants, FL-ΔH2 and FL-ΔH345, for their ability in PCP signaling. Since FL-ΔH2 expression does not rescue *ft* mutant animals to adulthood, our analysis was conducted in 3^rd^ instar eye discs. We found that the overall pattern of PCP is rescued when either FL-ΔH2 or FL-ΔH345 is expressed (Figure 4D, D’, G, G’). In each case, only a low rate of polarity errors was observed. This finding indicates that neither the H2 region nor the H345 region is essential for directing PCP in the eye. However, the somewhat elevated occurrence of polarity mistakes may suggest that the strength or quality of information is slightly reduced when the structure of the cytoplasmic domain is impaired. These results suggest that Ft regulates PCP through an effector region that is distinct from that used during growth control, and more importantly, that Ft’s roles in PCP signaling and tissue size control are separable and can be carried out independently. Consistent with this proposal, while MH2 sufficiently rescued *ft* animals to adulthood without restoring the normal pattern of planar cell polarity, expression of MH2 along with FL-ΔH2 can produce rescued adult animals with normal ommatidial organization (Figure 4H, H’).

### The Function of Ft to Regulate Tissue Size and Viability is Separable from its Ability to Regulate Clone Size and Hpo/Wts Signaling

**Figure 5:**
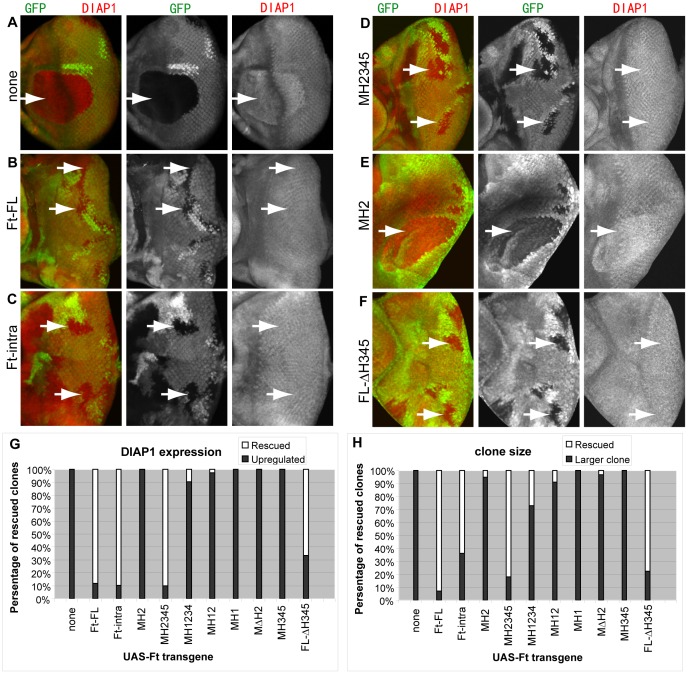
The H2 region is necessary but not sufficient for regulating the Hpo/Wts pathway. *ft^G−rv^* mutant clones, marked by the absence of GFP expression (green channel), were generated in third-instar eye imaginal discs in which Ft mutant transgenes were expressed by *TubP-Gal4*. DIAP1 staining was shown in red. White arrows point to *ft* mutant clones. (A) *ft* mutant clones show autonomous increases in DIAP1. Ubiquitous expression of Ft-FL (B), Ft-intra (C), MH2345 (D), and FL-ΔH345 (F) rescues the elevated DIAP1 levels in *ft* clones, whereas MH2 (E) does not rescue (see result section). (G) Quantification of the rescue of DIAP1 expression in *ft* clones. The plot shows the percentage of rescued clones observed with the indicated different Ft mutant transgenes. (H) Quantification of the rescue of clonal overgrowth in *ft* clones. The plot shows the percentage of rescued clones observed with the indicated different Ft mutant transgenes.

The requirement of Ft function in growth control can be seen in two different settings: the overall tissue size increase in the *ft* mutant animal and the clonal overgrowth in the mosaic tissue. Although expression of MH2 is sufficient to rescue tissue size and support viability in *ft* mutant animal, it surprisingly failed to rescue the clonal phenotype of *ft* mutant cells in the mosaic tissue (Figure 5E). In a more quantitative comparison, expression of the wild-type Ft (Ft-FL) restored the size and shape of *ft* clones to normal, *ft* mutant clones expressing MH2 are still large in size and round in shape, not significantly different from those lacking Ft function (Figure S2). These results suggest that the essential function of Ft for regulating viability and tissue size is separable from its regulation of clone size.

Several reports have demonstrated that the Hpo/Wts signaling pathway is a target of Ft regulation in growth control [17], [18], [19], [20]. This was most clearly shown in the mosaic animal in which clones of cells lacking Ft function have elevated Hpo/Wts signaling, resulting in the increase of clone size. The ability of Ft to regulate the Hpo/Wts pathway and the importance of the Hpo/Wts pathway for controlling cell proliferation and cell death has led to the idea that the essential function of Ft in supporting viability and preventing tissue size overgrowth is to stimulate the Hpo/Wts pathway and thus limit Yki activity.

Because we have observed that expression of MH2 provides sufficient Ft function to regulate tissue size and support viability without rescuing *ft* clonal overgrowth, this raises a question of whether expression of MH2 is able to restore Ft-mediated activation of the Hpo/Wts pathway. We first examined this question at the whole tissue level. Since it is difficult to compare the precise level of DIAP1 expression between eye discs by immunofluorescence, we used western blot to compare DIAP1 protein levels with β-tubulin as an internal control. While we observed a significant increase of DIAP1 in both *ft* mutant discs and MH2 rescued eye discs compared to *wt* discs, we could not detect significant difference of DIAP1 between *ft* mutant and MH2 rescued eye discs (Figure S2G, H).

We then tested this question at the clonal level by examining whether ubiquitous expression of MH2 prevents the elevated expression of Yki transcriptional targets, such as DIAP1, *fj-lacZ*, and CycE that would otherwise occur in clones of *ft* mutant cells (Figure 5). While ubiquitous expression of either Ft-FL or Ft-intra restored DIAP1 expression levels in *ft* clones to normal (Figures 5B, C), expression of MH2 appeared to have no effect on elevated DIAP1 expression in *ft* clones (Figure 5E). MH2 similarly failed to affect elevated CycE and *fj-lacZ* (Figure S3, S4). These results are consistent with the failure of MH2 to regulate clonal phenotype of *ft* mutant cells, suggesting that MH2 lacks the ability to restore Ft regulation of Hpo/Wts signaling.

**Figure 6:**
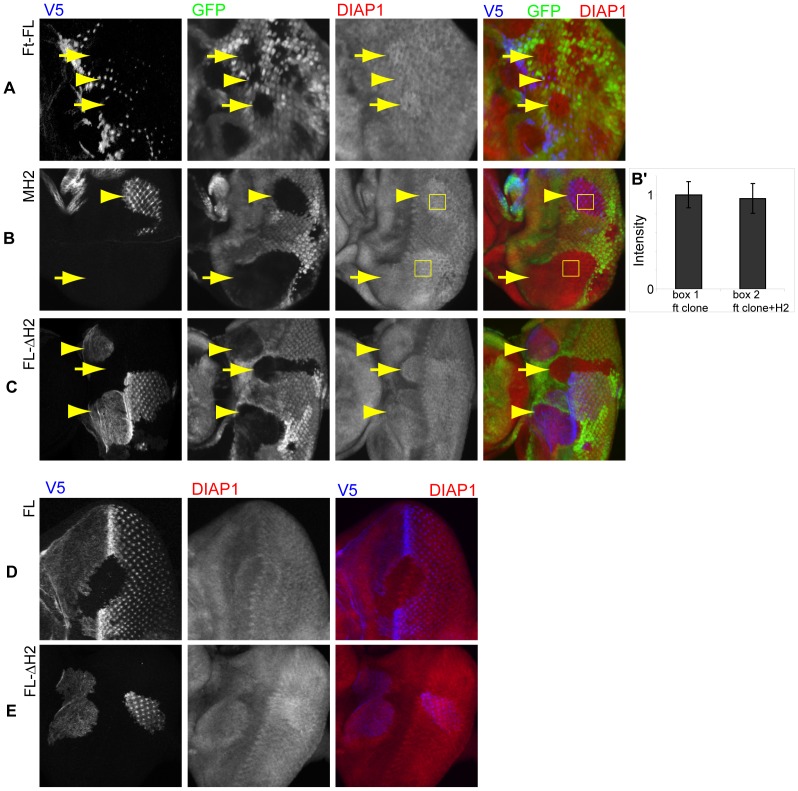
The H2 region has no detectable activity in the Hpo/Wts pathway. *ft* mutant clones, marked by the absence of GFP expression, were generated in third-instar eye imaginal discs, in which Ft mutant transgenes (blue): Ft-FL (A) and MH2 (B) were expressed after flip-out of *ActP>STOP, y^+^> Gal4*. Arrowheads point to *ft* mutant clones with expression of Ft transgenes. Arrows point to *ft* mutant clones alone. Eye imaginal discs were stained with GFP (green), DIAP1 (red), and V5 (blue). (A) Expression of Ft-FL suppressed elevated DIAP1 expression levels in *ft* clones. (B) Expression of MH2 failed to suppress elevated DIAP1 expression levels in *ft* clones. (B’) DIAP1 expression level in the *ft* clone was not significantly different from that of *ft* clone expressing MH2 in the same disc. DIAP1 expression level in the center of the clones (indicated in the yellow boxes in 6B) were quantitatively measured using histogram function of Adobe Photoshop, and normalized. n = 5. Two-tailed Student’s t-test. (C) Expression of FL-ΔH2 in *ft* clones did not suppress elevated DIAP1 expression levels. Cells in FL-ΔH2 expressing clones exhibited elevated levels of DIAP1 expression (E), whereas cells in Ft-FL (D) expressing clones did not have a dominant negative effect.

Since the ability to detect MH2 effect on the Hpo/Wts pathway may be limited by the need to make comparisons between discs, the possibility remained that MH2 might still regulate the Hpo/Wts pathway, albeit much more weakly than Ft-FL. In order to rigorously examine this possibility, we compared *ft* mutant clones that expressed MH2 to ones from the same eye disc that did not express MH2. For this experiment, *ft* mutant clones were generated in eye imaginal discs in which the flip-out GAL4 system had been used independently to generate independent clones of cells expressing MH2 under actin5C promoter control [50]. The resulting discs contained independent patches of *ft* mutant cells and MH2 expressing cells, which sometimes overlapped. As shown in Figure 6B and B’, the elevated DIAP1 level of *ft* mutant clones that expressed MH2 was quantitatively indistinguishable to that from *ft* mutant clones without expressing MH2. As expected, *ft* mutant clones that expressed Ft-FL effectively rescued DIAP1 expression level in this assay (Figure 6A). Together, these results indicate that the MH2 protein has little, if any, ability to activate the Hpo/Wts signaling pathway.

Interestingly, the protein lacking H2 region (FL-ΔH2) not only failed to suppress elevated DIAP1 expression levels in *ft* mutant clones, also induced dominant negative effect on Hpo/Wts signaling, as evident by the elevated expression of DIAP1 in wt cells overexpressing FL-ΔH2 (Figure 6C). This dominant negative effect was also apparent when clones of cells that overexpressed FL-ΔH2 were generated in otherwise wild-type animals (Figure 6E), indicating that FL-ΔH2 lacks the ability to provide the Hpo/Wts regulatory function, also dominantly inhibited the Hpo/Wts signaling ability of the endogenous wild-type Ft protein.

While the results described above demonstrate that expression of the H2 region alone is not sufficient to regulate Hpo/Wts signaling, they neither addressed whether the H2 region is required for this function nor identified other regions Ft that contribute to Hpo/Wts regulation. We investigated these issues by examining clonal size and DIAP1 expression in *ft* mutant clones generated in animals ubiquitously expressing various mutant forms of Ft proteins (Figure 5). Since most Ft mutant constructs cannot rescue the clonal phenotype and elevated DIAP1 expression, our interpretation is mainly focused on those with successful rescue. First, the expression of FL-ΔH345 effectively rescues Yki target gene expression and the *ft* clonal overgrowth phenotype, suggesting that the H345 region is not essential for this process. Second, the expression of Ft-intra and MH2345 effectively suppresses the enhanced expression of Yki targets in ft clones, suggesting the extracellular domain and the H1 region is not necessary. Third, the comparison between MH12 and FL-ΔH345, and the comparison between MH2345 and MH2 suggest that the presence of the extracellar domain and the H345 region may significantly enhance the signaling strength of the protein in regulating the Hpo/Wts pathway. Taken together, these results indicate that the H2 region, although not sufficient, is necessary for regulating the Hpo/Wts pathway, whereas H1, H345 and extracellular domain are not essential, but could potentially provide important supporting role for Hpo/Wts pathway.

## Discussion

### Separation of the Growth Control and PCP Function of Ft

The involvement of Ft in both tissue growth and PCP raises the question as to whether Ft regulates the two processes through the same or different regulatory domains. Here we identified two mutant proteins, and each harbors complementary properties. The H2 region is essential for tissue growth, but is completely dispensable for PCP signaling. The FL-ΔH2 protein lacks regulatory function for tissue growth but retains full function in PCP. This strongly indicates that roles of Ft in tissue growth regulation and PCP signaling are independent, and each can be carried out by a separate effector region.

There are several reported binding partners of Ft cytoplasmic domain, which have been reported to mediate some function of Fat [40], [41], [42], [43], [57]. The key interaction regions are indicated in Figure 1. However, these regions are not essential for Ft function in tissue size, PCP and Hpo/Wts activity. This is best shown by one mutant Ft protein, FL-ΔH345, which lacks the reported binding regions, but is still sufficient to rescue Ft functions in these processes. This result indicates that these binding partners are not likely the primary effectors, but modulators in mediating downstream signaling.

### Different Requirement for Ft Function in PCP Signaling between the Eye and Wing

In the eye, we showed that the crucial region of Ft for PCP signaling includes the extracellular domain. In the absence of the extracellular domain, the eye displays severe PCP defects. This result is consistent with the model in previous *in vivo* and *in vitro* studies that Ft and Ds can form a heterodimer through their extracellular domains and this interaction is essential for the ordered PCP pattern [1], [45]. Second, this result draws a clear distinction between the eye and wing. In the wing, the transmembrane and cytoplasmic domains of Ft are sufficient to rescue the PCP defect in the wing, suggesting this protein contains all the effector regions for PCP signaling [52]. Since the extracellular domain of Ft is required to interact with Ds to receive the upstream directional information in the eye, the dispensability of the extracelluar domain during the polarization of the wing indicates that this directional information may not be essential in the wing. This is consistent with the result shown in a previous study that the Ds and Fj gradients are essential for the eye, but do not play a major role in directing PCP in the wing [3]. It is worth pointing out that the dispensability of Ds and Fj gradients is NOT equivalent to the removal of Ds function, as Ds has an essential function to stabilize and/or activate Ft on the plasma membrane in addition to its role of providing a directional cue. Together, these observations suggest a model in which Ft needs Ds to activate its PCP function; and both the activation and its graded fashion across the tissue are required in the eye to provide a directional cue, whereas in the wing this activation is not necessary to be graded. Thus, this study supports the notion that the eye and wing have different requirement for Ft function, indicating different strategies used to direct PCP.

### Ft’s Role in Tissue Size Regulation is Genetically Separable from its Function in Clone Size Regulation

Role of Ft in regulating growth can often be revealed in two different settings. First, animals lacking Ft function exhibit massive overgrowth of all imaginal discs. Second, in mosaic animals, cells lacking Ft function proliferate much more extensively than their wild-type neighbors, resulting in large clones of mutant cells. While each of these observations suggests that cells lacking Ft are unable to respond to signals that normally limit their growth and proliferation, they may reflect different roles of Ft. The increase of clone size may result from mutant cells possessing a competitive advantage over their neighbors, such as an increased rate of proliferation or resistance to cell death. In contrast, the imaginal disc overgrowth in animals lacking Ft function may indicate that *ft* mutant cells are unable to sense or respond to the signals which normally restrain tissue growth and thus help specify tissue size. Although these two properties can often be affected by the same cellular events, they are not always correlated. In this study, we indeed observed the dissociation of these two aspects of Ft function. This is most clearly shown by MH2, which is sufficient to rescue tissue size overgrowth but lacking the ability to rescue clonal overgrowth defect.

One downstream pathway of Fat is to stimulate the activity of the Hpo/Wts signaling pathway, thus leading to inhibition of the transcriptional activator Yki and reduced expression of its targets [14], [36]. These targets include important positive regulators of growth and negative regulators of apoptosis such as CycE and DIAP1. The ability of Ft to regulate the Hpo/Wts pathway and the similar clonal overgrowth phenotypes of animals lacking either Ft or Hpo/Wts pathway activity has led to the idea that the essential function of Ft in supporting viability and preventing tissue overgrowth is to activate the Hpo/Wts pathway. Indeed, genetic interactions between *ft* and the Hpo/Wts pathway are consistent with this idea. For example, the imaginal disc overgrowth phenotype of *ft* mutant animals is partially suppressed and enhanced by heterozygosity for Yki and Wts mutations, respectively [20].

Given the strength of these arguments, our expectation was that the minimal region of Ft required for regulating tissue size and supporting viability would also be capable of regulating the expression of Yki targets. Surprisingly, our analysis demonstrates that the ability to regulate tissue size and viability does not correlate with the ability to repress Yki activity. This is most clearly seen in the ability of the H2 region, when membrane-tagged, to rescue viability and tissue size without having any detectable ability to regulate Yki target expression. Thus, these data argue that the essential function of Ft in viability and tissue size regulation is genetically separable from its role in Hpo/Wts pathway regulation. In agreement with our observation, a previous study has reported growth defect in *ft* mutant tissue that cannot be explained by loss of *expanded (ex)*, an upstream component of the Hpo/Wts pathway, suggesting that there are other, as-yet-unidentified, mediators of *ft* [18].

Previous research has shown that regulation of tissue growth and proliferation by Ft is complex [17], [18], [19], [20], [36], [58], [59]. The complex nature of the signaling network may reflect multiple levels of regulation in tissue growth and proliferation control. It is possible that multiple mechanisms at different levels regulate distinct aspects of tissue growth. Indeed, our studies support such a model. During the control of tissue growth and proliferation, Hpo/Wts signaling may serve primarily as a local regulatory mechanism to coordinate cell proliferation and cell death among neighboring cells, whereas an unidentified pathway mediated by Ft may play a more crucial role in allowing the tissue as whole to regulate its size. Although not favored by our data, the possibility cannot be excluded that dissociation of tissue size control from clone size regulation is due to quantitative difference in Hpo/Wts signaling. However, within the sensitivity range of all the *in vivo* assays used in this study, we could not detect any activity of MH2 in regulating the Hpo/Wts pathway. Therefore, our results are more consistent with the model in which an unknown pathway mediated by Ft regulates tissue size. To completely understand how Ft fulfills these roles and the underlying biochemical mechanisms will likely require the identification of key binding partners for the crucial H2 region.

While this manuscript was under preparation, a similar structure-function analysis was published based on the analysis of Ft function in the wing [44]. In agreement with our analysis, Matakatsu et al. have found a region (termed Hpo-active domain) required for viability, tissue size regulation and Hpo/Wts signaling activity, which largely overlaps with the H2 region. The study from Matakatsu et al. did not report any mutant construct that can clearly rescue viability but not activate the Hpo/Wts signaling pathway. However, we have observed such phenomenon that expression of the H2 region is not sufficient to activate Hpo/Wts signaling in our assays even though the H2 region is fully capable to support viability and limit tissue size. Several differences in the experimental set up between the two studies may contribute to the difference in observation. Besides the tissue difference between the wing and eye, the two studies used different version of the truncation mutants of Ft, different pathway readouts for Hpo/Wts signaling, different Gal4 drivers, and different tissue locations to examine the effect on growth and Hpo/Wts signaling. In the future, it will be interesting to test whether such phenomenon can be observed across different tissues. Comparing to the required regions for PCP in the wing, we have demonstrated that the extracellular domain of Ft is essential for PCP in the eye. This difference reflects different strategies to organize PCP between eye and wing [3], [45], [52]. Finally, both studies support the idea that the functional region for PCP is distinct from the region for tissue growth regulation.

During the revision of this manuscript, another structure-function study of Ft was published based on the analysis in the wing and abdomen [60]. Using a complementary approach - small deletion or point mutations in *ft* genomic constructs rather than UAS-Gal4 system, Pan et al. also reached the same conclusion that effects of Ft on wing growth versus PCP can be separated at the level of Ft itself. Interestingly, one unique finding from Pan et al. indicates a 4 amino acids C-terminal motif that is essential for Ft-mediated PCP in certain regions of the wing and abdomen. Intriguingly, we found the mutant Ft without C-terminal region (FL-ΔH345) is still capable of directing PCP in the eye with a fidelity rate of 91.1%. In the future, it would be interesting to exam how much of the reduced fidelity rate of FL-ΔH345 in the eye is because of missing this 4 amino acids motif.

## Supporting Information

Figure S1 **Ft regulates tissue size through a tissue-autonomous manner.** Expression of either Ft-FL or Ft-intra under Eyeless-Gal4 control restore eye imaginal disc size to normal, but not that of other tissues, such as wing discs. (A–D) Third-instar eye imaginal discs stained with fluorescein phalloidin. (A’–D’) Third-instar wing imaginal discs. Genotypes are: (A, A’) *wt*, (B, B’) *ft−/−*, (C, C’) *ft−/−; Ey-Gal4/UAS-Ft-FL*, (D, D’) *ft−/−; Ey-Gal4/UAS-Ft-intra*
(PDF)

Figure S2 ***ft*** **mutant clones expressing MH2 are still large in size and round in shape, similar to those lacking Ft function.** (A–D) Examples of clone/twin-spot pairs showing increased size and round shape of *ft* mutant clone compared to its twin-spot. This clonal phenotype is fully rescued by the ubiquitous expression of Ft-FL, but not significantly affected by the expression of MH2. (A) *wt* clones (marked by loss of GFP, indicated by white arrow) are similar in size and shape to their twin spot (GFP/GFP, indicated by yellow arrow). (B) *ft* clones (marked by loss of GFP, indicated by white arrow) are larger than their twin spot (indicated by yellow arrow) in larval eye discs. (C) Ubiquitous expression of wild-type Ft (Ft-FL) with *TubP-Gal4* restored the size and shape of *ft* clones to normal. (D) *ft* clones expressing MH2 retained the large size and round shape. (E) Quantification of the ratio of clone size/twin spot. Clone and twin spot areas were measured using the histogram function of Adobe photoshop. Black bar represents average ratio. Average ratio was 0.94 for *wt*, 6.56 for *ft* clone, 0.85 for *ft* clone+Ft-FL and 5.94 for *ft* clone+H2. (F) Quantification of the ratio of clone shape index/twin spot. Shape of each individual clone or twin spot was determined by clone shape index (A/L^2^) of roundness (L: circumference of the clone, A: area of the clone). The rational for using A/L^2^ formula was as follows: for a given number of cells in a clone, the stronger the tendency of these cells to avoid mixing with cells located outside of the clone, the smaller the circumference (L) would be relative to the area (A) [61], and the larger the clone shape index (A/L^2^). The area (A) and the circumference (L) of a clone or twin spot were measured using the histogram function of Adobe photoshop. Black bar represents average ratio. Average ratio was 1.12 for *wt*, 2.89 for *ft* clone, 1.04 for *ft* clone+Ft-FL and 2.40 for *ft* clone+H2. (GH) Immunoblot analysis of DIAP1 expression in eye imaginal disc extracts from *wt, ft* mutant, and *ft* mutant animals expressing Ft-FL or MH2 transgenes. Blots were probed with anti-DIAP1 and anti-β-tubulin. DIAP1 expression levels in the *ft* mutant and MH2 rescued discs are significantly increased compared to the *wt* and Ft-FL rescued discs (p<0.05). No significant difference can be detected between the *ft* mutant and MH2 rescued discs. DIAP1 levels were quantified and normalized to the value in *wt* animals. An example of immunoblot is shown in (G). Quantification result in (H) represents the mean of three independent experiments and error bars show S.D. Statistical significance was analyzed by one-way ANOVA with post-hoc bonferroni correction.
(PDF)

Figure S3 **The H2 region is not sufficient to rescue elevated Cyclin E expression in** ***ft*** **clones.** *ft^G−rv^* mutant clones, marked by the absence of GFP expression (green channel), were generated in third-instar eye imaginal discs in which Ft mutant transgenes were expressed by *TubP-Gal4*. Cyclin E staining was shown in red. (A) *ft* mutant clones show autonomous increases in Cyclin E. Ubiquitous expression of Ft-FL (B), and Ft-intra (C) rescues the elevated Cyclin E levels in *ft* clones, whereas MH2 (D) and MΔH2 (E) do not rescue (see result section).
(PDF)

Figure S4 **The H2 region is not sufficient to rescue elevated** ***fj-lacZ*** **expression in** ***ft*** **clones.** *ft^G−rv^* mutant clones, marked by the absence of GFP expression (green channel), were generated in third-instar eye imaginal discs in which Ft mutant transgenes were expressed by *TubP-Gal4*. β-gal staining was shown in red. (A) *ft* mutant clones show autonomous increases in *fj-lacZ*. Ubiquitous expression of Ft-FL (B), and Ft-intra (C) rescues the elevated *fj-lacZ* levels in *ft* clones, whereas MH2 (D) and MΔH2 (E) do not rescue (see result section).
(PDF)

## References

[1] Ma D, Yang CH, McNeill H, Simon MA, Axelrod JD (2003) Fidelity in planar cell polarity signalling. Nature 421: 543–547. 12540853 10.1038/nature01366

[2] Yang CH, Axelrod JD, Simon MA (2002) Regulation of Frizzled by fat-like cadherins during planar polarity signaling in the Drosophila compound eye. Cell 108: 675–688. 11893338 10.1016/s0092-8674(02)00658-x

[3] Simon MA (2004) Planar cell polarity in the Drosophila eye is directed by graded Four-jointed and Dachsous expression. Development 131: 6175–6184. 15548581 10.1242/dev.01550

[4] Tree DR, Ma D, Axelrod JD (2002) A three-tiered mechanism for regulation of planar cell polarity. Semin Cell Dev Biol 13: 217–224. 12137730 10.1016/s1084-9521(02)00042-3

[5] Lawrence PA, Struhl G, Casal J (2007) Planar cell polarity: one or two pathways? Nat Rev Genet 8: 555–563. 17563758 10.1038/nrg2125 PMC2747024

[6] Axelrod JD (2009) Progress and challenges in understanding planar cell polarity signaling. Semin Cell Dev Biol 20: 964–971. 19665570 10.1016/j.semcdb.2009.08.001

[7] Bayly R, Axelrod JD (2011) Pointing in the right direction: new developments in the field of planar cell polarity. Nat Rev Genet 12: 385–391. 21502960 10.1038/nrg2956 PMC4854751

[8] Strutt D (2009) Gradients and the specification of planar polarity in the insect cuticle. Cold Spring Harb Perspect Biol 1: a000489. 20066114 10.1101/cshperspect.a000489 PMC2773641

[9] Thomas C, Strutt D (2012) The roles of the cadherins Fat and Dachsous in planar polarity specification in Drosophila. Dev Dyn 241: 27–39. 21919123 10.1002/dvdy.22736

[10] Mahoney PA, Weber U, Onofrechuk P, Biessmann H, Bryant PJ, et al (1991) The fat tumor suppressor gene in Drosophila encodes a novel member of the cadherin gene superfamily. Cell 67: 853–868. 1959133 10.1016/0092-8674(91)90359-7

[11] Bryant PJ, Huettner B, Held LI Jr, Ryerse J, Szidonya J (1988) Mutations at the fat locus interfere with cell proliferation control and epithelial morphogenesis in Drosophila. Dev Biol 129: 541–554. 3417051 10.1016/0012-1606(88)90399-5

[12] Garoia F, Guerra D, Pezzoli MC, Lopez-Varea A, Cavicchi S, et al (2000) Cell behaviour of Drosophila fat cadherin mutations in wing development. Mech Dev 94: 95–109. 10842062 10.1016/s0925-4773(00)00306-3

[13] Schroeder MC, Halder G (2012) Regulation of the Hippo pathway by cell architecture and mechanical signals. Semin Cell Dev Biol. 10.1016/j.semcdb.2012.06.001 22750148

[14] Pan D (2010) The hippo signaling pathway in development and cancer. Dev Cell 19: 491–505. 20951342 10.1016/j.devcel.2010.09.011 PMC3124840

[15] Harvey KF, Hariharan IK (2012) The Hippo Pathway. Cold Spring Harb Perspect Biol. 10.1101/cshperspect.a011288 PMC4413430 22745287

[16] Staley BK, Irvine KD (2012) Hippo signaling in Drosophila: recent advances and insights. Dev Dyn 241: 3–15. 22174083 10.1002/dvdy.22723 PMC3426292

[17] Willecke M, Hamaratoglu F, Kango-Singh M, Udan R, Chen CL, et al (2006) The fat cadherin acts through the hippo tumor-suppressor pathway to regulate tissue size. Curr Biol 16: 2090–2100. 16996265 10.1016/j.cub.2006.09.005

[18] Silva E, Tsatskis Y, Gardano L, Tapon N, McNeill H (2006) The tumor-suppressor gene fat controls tissue growth upstream of expanded in the hippo signaling pathway. Curr Biol 16: 2081–2089. 16996266 10.1016/j.cub.2006.09.004

[19] Cho E, Feng Y, Rauskolb C, Maitra S, Fehon R, et al (2006) Delineation of a Fat tumor suppressor pathway. Nat Genet 38: 1142–1150. 16980976 10.1038/ng1887

[20] Bennett FC, Harvey KF (2006) Fat cadherin modulates organ size in Drosophila via the Salvador/Warts/Hippo signaling pathway. Curr Biol 16: 2101–2110. 17045801 10.1016/j.cub.2006.09.045

[21] Tyler DM, Baker NE (2007) Expanded and fat regulate growth and differentiation in the Drosophila eye through multiple signaling pathways. Dev Biol 305: 187–201. 17359963 10.1016/j.ydbio.2007.02.004 PMC2075468

[22] Harvey KF, Pfleger CM, Hariharan IK (2003) The Drosophila Mst ortholog, hippo, restricts growth and cell proliferation and promotes apoptosis. Cell 114: 457–467. 12941274 10.1016/s0092-8674(03)00557-9

[23] Pantalacci S, Tapon N, Leopold P (2003) The Salvador partner Hippo promotes apoptosis and cell-cycle exit in Drosophila. Nat Cell Biol 5: 921–927. 14502295 10.1038/ncb1051

[24] Udan RS, Kango-Singh M, Nolo R, Tao C, Halder G (2003) Hippo promotes proliferation arrest and apoptosis in the Salvador/Warts pathway. Nat Cell Biol 5: 914–920. 14502294 10.1038/ncb1050

[25] Wu S, Huang J, Dong J, Pan D (2003) hippo encodes a Ste-20 family protein kinase that restricts cell proliferation and promotes apoptosis in conjunction with salvador and warts. Cell 114: 445–456. 12941273 10.1016/s0092-8674(03)00549-x

[26] Jia J, Zhang W, Wang B, Trinko R, Jiang J (2003) The Drosophila Ste20 family kinase dMST functions as a tumor suppressor by restricting cell proliferation and promoting apoptosis. Genes Dev 17: 2514–2519. 14561774 10.1101/gad.1134003 PMC218145

[27] Xu T, Wang W, Zhang S, Stewart RA, Yu W (1995) Identifying tumor suppressors in genetic mosaics: the Drosophila lats gene encodes a putative protein kinase. Development 121: 1053–1063. 7743921 10.1242/dev.121.4.1053

[28] Justice RW, Zilian O, Woods DF, Noll M, Bryant PJ (1995) The Drosophila tumor suppressor gene warts encodes a homolog of human myotonic dystrophy kinase and is required for the control of cell shape and proliferation. Genes Dev 9: 534–546. 7698644 10.1101/gad.9.5.534

[29] Kango-Singh M, Nolo R, Tao C, Verstreken P, Hiesinger PR, et al (2002) Shar-pei mediates cell proliferation arrest during imaginal disc growth in Drosophila. Development 129: 5719–5730. 12421711 10.1242/dev.00168

[30] Tapon N, Harvey KF, Bell DW, Wahrer DC, Schiripo TA, et al (2002) salvador Promotes both cell cycle exit and apoptosis in Drosophila and is mutated in human cancer cell lines. Cell 110: 467–478. 12202036 10.1016/s0092-8674(02)00824-3

[31] Lai ZC, Wei X, Shimizu T, Ramos E, Rohrbaugh M, et al (2005) Control of cell proliferation and apoptosis by mob as tumor suppressor, mats. Cell 120: 675–685. 15766530 10.1016/j.cell.2004.12.036

[32] Huang J, Wu S, Barrera J, Matthews K, Pan D (2005) The Hippo signaling pathway coordinately regulates cell proliferation and apoptosis by inactivating Yorkie, the Drosophila Homolog of YAP. Cell 122: 421–434. 16096061 10.1016/j.cell.2005.06.007

[33] Dong J, Feldmann G, Huang J, Wu S, Zhang N, et al (2007) Elucidation of a universal size-control mechanism in Drosophila and mammals. Cell 130: 1120–1133. 17889654 10.1016/j.cell.2007.07.019 PMC2666353

[34] Oh H, Irvine KD (2008) In vivo regulation of Yorkie phosphorylation and localization. Development 135: 1081–1088. 18256197 10.1242/dev.015255 PMC2387210

[35] Hariharan IK, Bilder D (2006) Regulation of imaginal disc growth by tumor-suppressor genes in Drosophila. Annu Rev Genet 40: 335–361. 16872256 10.1146/annurev.genet.39.073003.100738

[36] Hariharan IK (2006) Growth regulation: a beginning for the hippo pathway. Curr Biol 16: R1037–1039. 17174912 10.1016/j.cub.2006.11.012

[37] Edgar BA (2006) From cell structure to transcription: Hippo forges a new path. Cell 124: 267–273. 16439203 10.1016/j.cell.2006.01.005

[38] Matakatsu H, Blair SS (2008) The DHHC palmitoyltransferase approximated regulates Fat signaling and Dachs localization and activity. Curr Biol 18: 1390–1395. 18804377 10.1016/j.cub.2008.07.067 PMC2597019

[39] Mao Y, Rauskolb C, Cho E, Hu WL, Hayter H, et al (2006) Dachs: an unconventional myosin that functions downstream of Fat to regulate growth, affinity and gene expression in Drosophila. Development 133: 2539–2551. 16735478 10.1242/dev.02427

[40] Fanto M, Clayton L, Meredith J, Hardiman K, Charroux B, et al (2003) The tumor-suppressor and cell adhesion molecule Fat controls planar polarity via physical interactions with Atrophin, a transcriptional co-repressor. Development 130: 763–774. 12506006 10.1242/dev.00304

[41] Mao Y, Kucuk B, Irvine KD (2009) Drosophila lowfat, a novel modulator of Fat signaling. Development 136: 3223–3233. 19710173 10.1242/dev.036152 PMC2739141

[42] Feng Y, Irvine KD (2009) Processing and phosphorylation of the Fat receptor. Proceedings of the National Academy of Sciences 106: 11989–11994. 10.1073/pnas.0811540106 PMC2709664 19574458

[43] Sopko R, Silva E, Clayton L, Gardano L, Barrios-Rodiles M, et al (2009) Phosphorylation of the Tumor Suppressor Fat Is Regulated by Its Ligand Dachsous and the Kinase Discs Overgrown. Current Biology 19: 1112–1117. 19540118 10.1016/j.cub.2009.05.049 PMC2851237

[44] Matakatsu H, Blair SS (2012) Separating planar cell polarity and Hippo pathway activities of the protocadherins Fat and Dachsous. Development 139: 1498–1508. 22399682 10.1242/dev.070367 PMC3308182

[45] Matakatsu H, Blair SS (2004) Interactions between Fat and Dachsous and the regulation of planar cell polarity in the Drosophila wing. Development 131: 3785–3794. 15240556 10.1242/dev.01254

[46] Cooper MT, Bray SJ (1999) Frizzled regulation of Notch signalling polarizes cell fate in the Drosophila eye. Nature 397: 526–530. 10028969 10.1038/17395

[47] Buckles GR, Rauskolb C, Villano JL, Katz FN (2001) Four-jointed interacts with dachs, abelson and enabled and feeds back onto the Notch pathway to affect growth and segmentation in the Drosophila leg. Development 128: 3533–3542. 11566858 10.1242/dev.128.18.3533

[48] Lee T, Luo L (2001) Mosaic analysis with a repressible cell marker (MARCM) for Drosophila neural development. Trends Neurosci 24: 251–254. 11311363 10.1016/s0166-2236(00)01791-4

[49] Tang CY, Sun YH (2002) Use of mini-white as a reporter gene to screen for GAL4 insertions with spatially restricted expression pattern in the developing eye in drosophila. Genesis 34: 39–45. 12324945 10.1002/gene.10135

[50] Struhl G, Fitzgerald K, Greenwald I (1993) Intrinsic activity of the Lin-12 and Notch intracellular domains in vivo. Cell 74: 331–345. 8343960 10.1016/0092-8674(93)90424-o

[51] Xu T, Rubin GM (1993) Analysis of genetic mosaics in developing and adult Drosophila tissues. Development 117: 1223–1237. 8404527 10.1242/dev.117.4.1223

[52] Matakatsu H, Blair SS (2006) Separating the adhesive and signaling functions of the Fat and Dachsous protocadherins. Development 133: 2315–2324. 16687445 10.1242/dev.02401

[53] Brand AH, Perrimon N (1993) Targeted gene expression as a means of altering cell fates and generating dominant phenotypes. Development 118: 401–415. 8223268 10.1242/dev.118.2.401

[54] Rorth P (1998) Gal4 in the Drosophila female germline. Mech Dev 78: 113–118. 9858703 10.1016/s0925-4773(98)00157-9

[55] Struhl G, Adachi A (1998) Nuclear Access and Action of Notch In Vivo. Cell 93: 649–660. 9604939 10.1016/s0092-8674(00)81193-9

[56] Tomlinson A, Ready DF (1987) Cell fate in the Drosophila ommatidium. Developmental Biology 123: 264–275. 17985474 10.1016/0012-1606(87)90448-9

[57] Ishiuchi T, Misaki K, Yonemura S, Takeichi M, Tanoue T (2009) Mammalian Fat and Dachsous cadherins regulate apical membrane organization in the embryonic cerebral cortex. J Cell Biol 185: 959–967. 19506035 10.1083/jcb.200811030 PMC2711618

[58] Tyler D, Li W, Zhuo N, Pellock BJ, Baker NE (2006) Genes affecting cell competition in Drosophila melanogaster. Genetics. 10.1534/genetics.106.061929 PMC1800612 17110495

[59] Feng Y, Irvine KD (2007) Fat and expanded act in parallel to regulate growth through warts. Proc Natl Acad Sci U S A 104: 20362–20367. 18077345 10.1073/pnas.0706722105 PMC2154436

[60] Pan G, Feng Y, Ambegaonkar AA, Sun G, Huff M, et al (2013) Signal transduction by the Fat cytoplasmic domain. Development 140: 831–842. 23318637 10.1242/dev.088534 PMC3557778

[61] Lawrence PA, Casal J, Struhl G (1999) The hedgehog morphogen and gradients of cell affinity in the abdomen of Drosophila. Development 126: 2441–2449. 10226003 10.1242/dev.126.11.2441

