# Supplementary figures and images for: The Drosophila Cadherin Fat Regulates Tissue Size and Planar Cell Polarity through Different Domains
Source: PLoS One. 2013 May 7;8(5):e62998. doi: 10.1371/journal.pone.0062998 (PMC3647076; doi:10.1371/journal.pone.0062998)

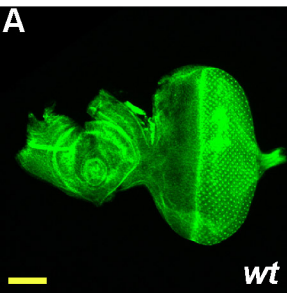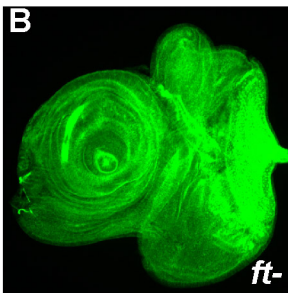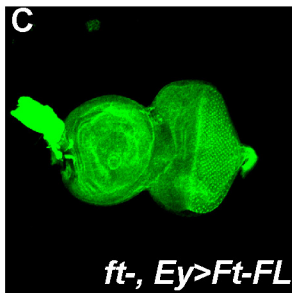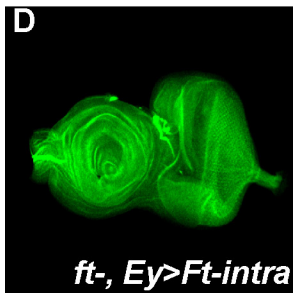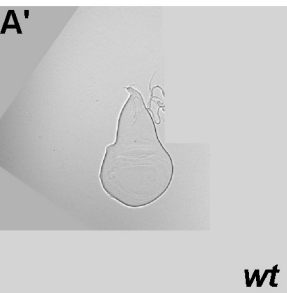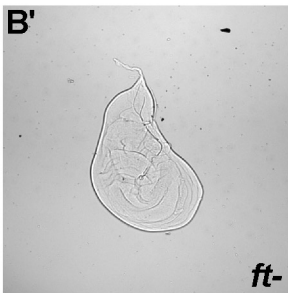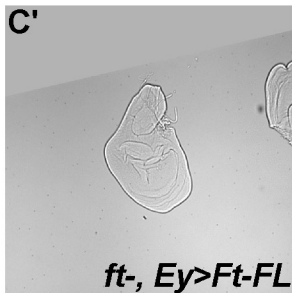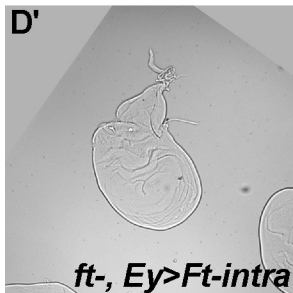

Supplement: Figure S1 — Ft regulates tissue size through a tissue-autonomous manner. Expression of either Ft-FL or Ft-intra under Eyeless-Gal4 control restore eye imaginal disc size to normal, but not that of other tissues, such as wing discs. (A–D) Third-instar eye imaginal discs stained with fluorescein phalloidin. (A’–D’) Third-instar wing imaginal discs. Genotypes are: (A, A’) wt, (B, B’) ft−/−, (C, C’) ft−/−; Ey-Gal4/UAS-Ft-FL, (D, D’) ft−/−; Ey-Gal4/UAS-Ft-intra (PDF) [file pone.0062998.s001.pdf]

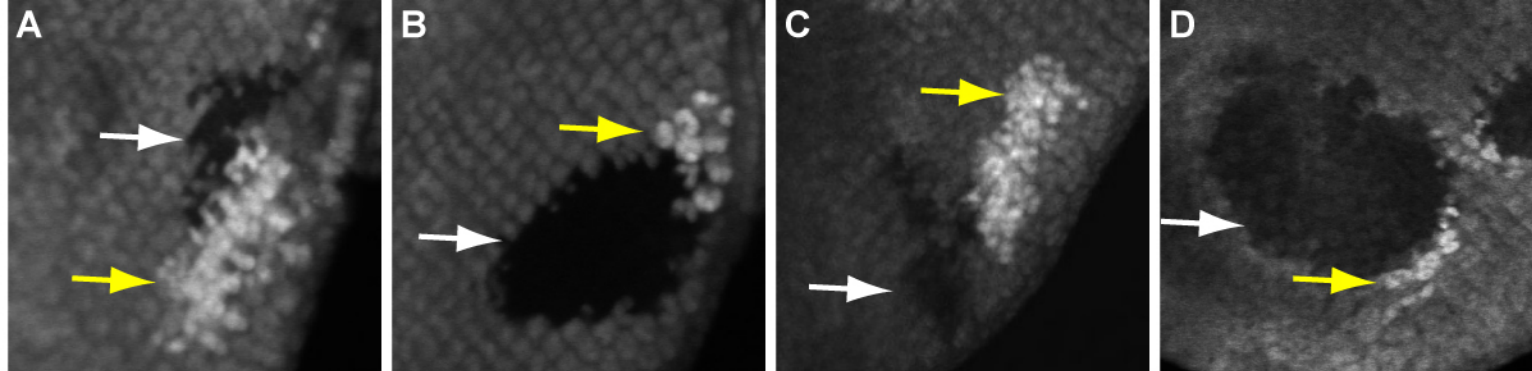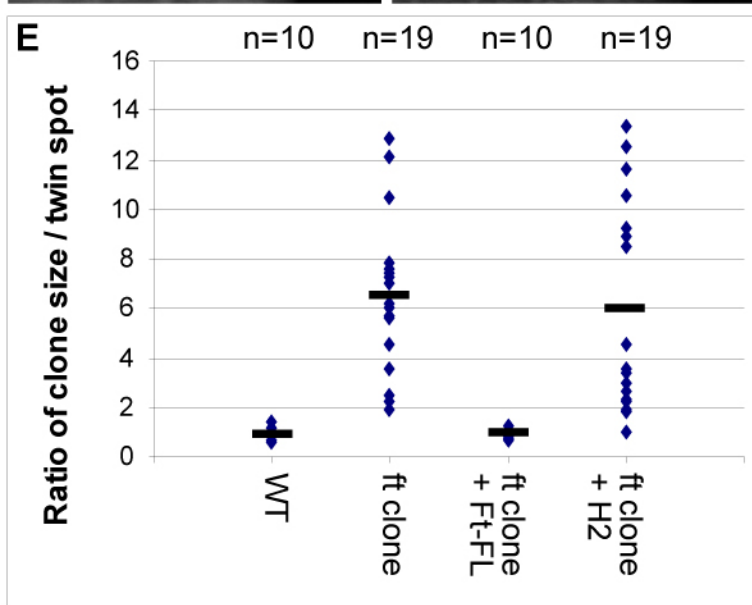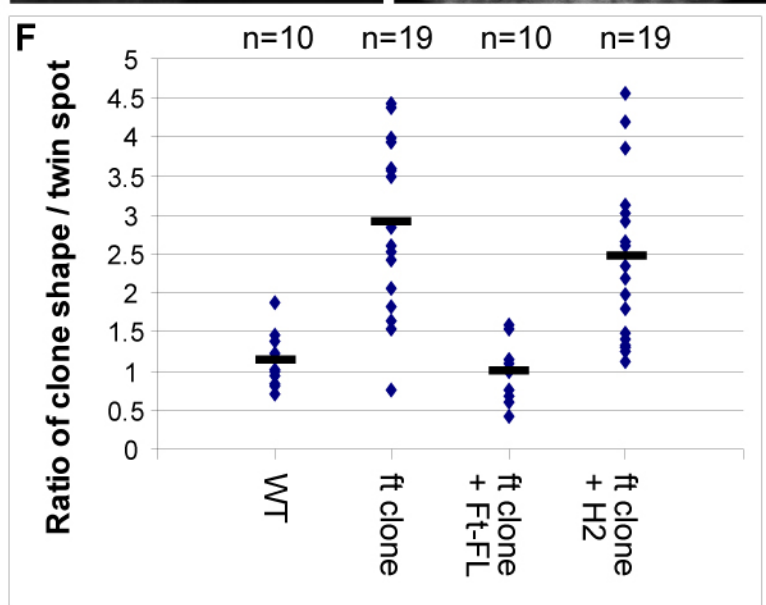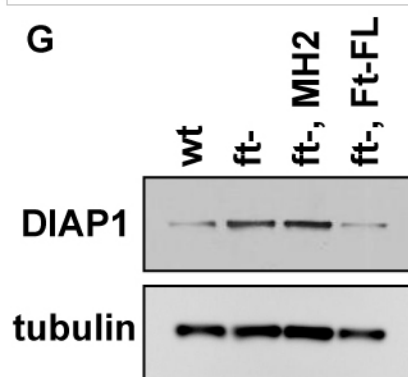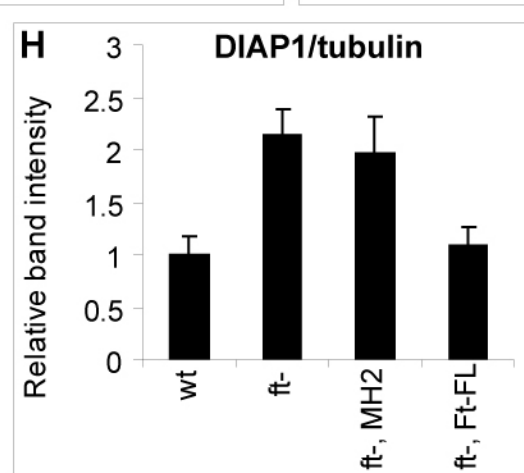

Supplement: Figure S2 — ft mutant clones expressing MH2 are still large in size and round in shape, similar to those lacking Ft function. (A–D) Examples of clone/twin-spot pairs showing increased size and round shape of ft mutant clone compared to its twin-spot. This clonal phenotype is fully rescued by the ubiquitous expression of Ft-FL, but not significantly affected by the expression of MH2. (A) wt clones (marked by loss of GFP, indicated by white arrow) are similar in size and shape to their twin spot (GFP/GFP, indicated by yellow arrow). (B) ft clones (marked by loss of GFP, indicated by white arrow) are larger than their twin spot (indicated by yellow arrow) in larval eye discs. (C) Ubiquitous expression of wild-type Ft (Ft-FL) with TubP-Gal4 restored the size and shape of ft clones to normal. (D) ft clones expressing MH2 retained the large size and round shape. (E) Quantification of the ratio of clone size/twin spot. Clone and twin spot areas were measured using the histogram function of Adobe photoshop. Black bar represents average ratio. Average ratio was 0.94 for wt, 6.56 for ft clone, 0.85 for ft clone+Ft-FL and 5.94 for ft clone+H2. (F) Quantification of the ratio of clone shape index/twin spot. Shape of each individual clone or twin spot was determined by clone shape index (A/L2) of roundness (L: circumference of the clone, A: area of the clone). The rational for using A/L2 formula was as follows: for a given number of cells in a clone, the stronger the tendency of these cells to avoid mixing with cells located outside of the clone, the smaller the circumference (L) would be relative to the area (A) [61], and the larger the clone shape index (A/L2). The area (A) and the circumference (L) of a clone or twin spot were measured using the histogram function of Adobe photoshop. Black bar represents average ratio. Average ratio was 1.12 for wt, 2.89 for ft clone, 1.04 for ft clone+Ft-FL and 2.40 for ft clone+H2. (GH) Immunoblot analysis of DIAP1 expression in eye imaginal disc extra [file pone.0062998.s002.pdf]

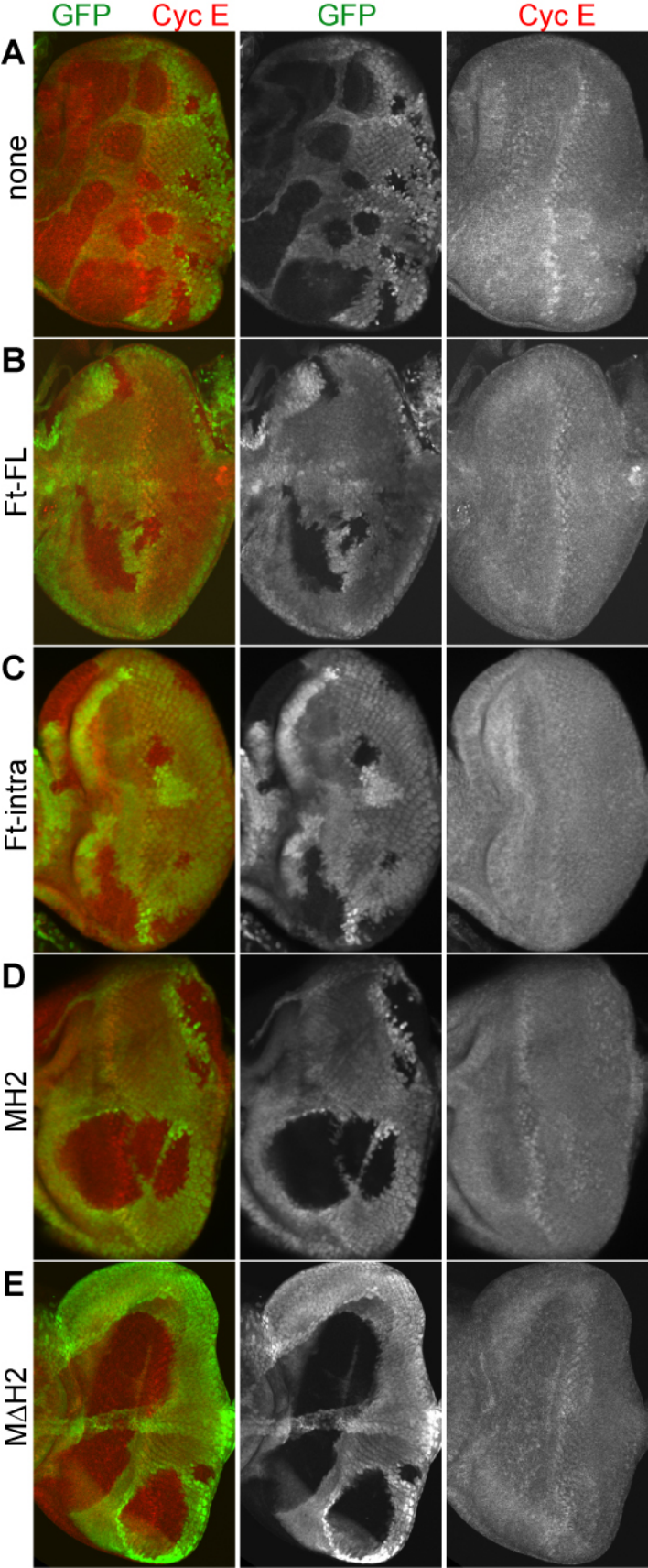

Supplement: Figure S3 — The H2 region is not sufficient to rescue elevated Cyclin E expression in ft clones. ftG−rv mutant clones, marked by the absence of GFP expression (green channel), were generated in third-instar eye imaginal discs in which Ft mutant transgenes were expressed by TubP-Gal4. Cyclin E staining was shown in red. (A) ft mutant clones show autonomous increases in Cyclin E. Ubiquitous expression of Ft-FL (B), and Ft-intra (C) rescues the elevated Cyclin E levels in ft clones, whereas MH2 (D) and MΔH2 (E) do not rescue (see result section). (PDF) [file pone.0062998.s003.pdf]

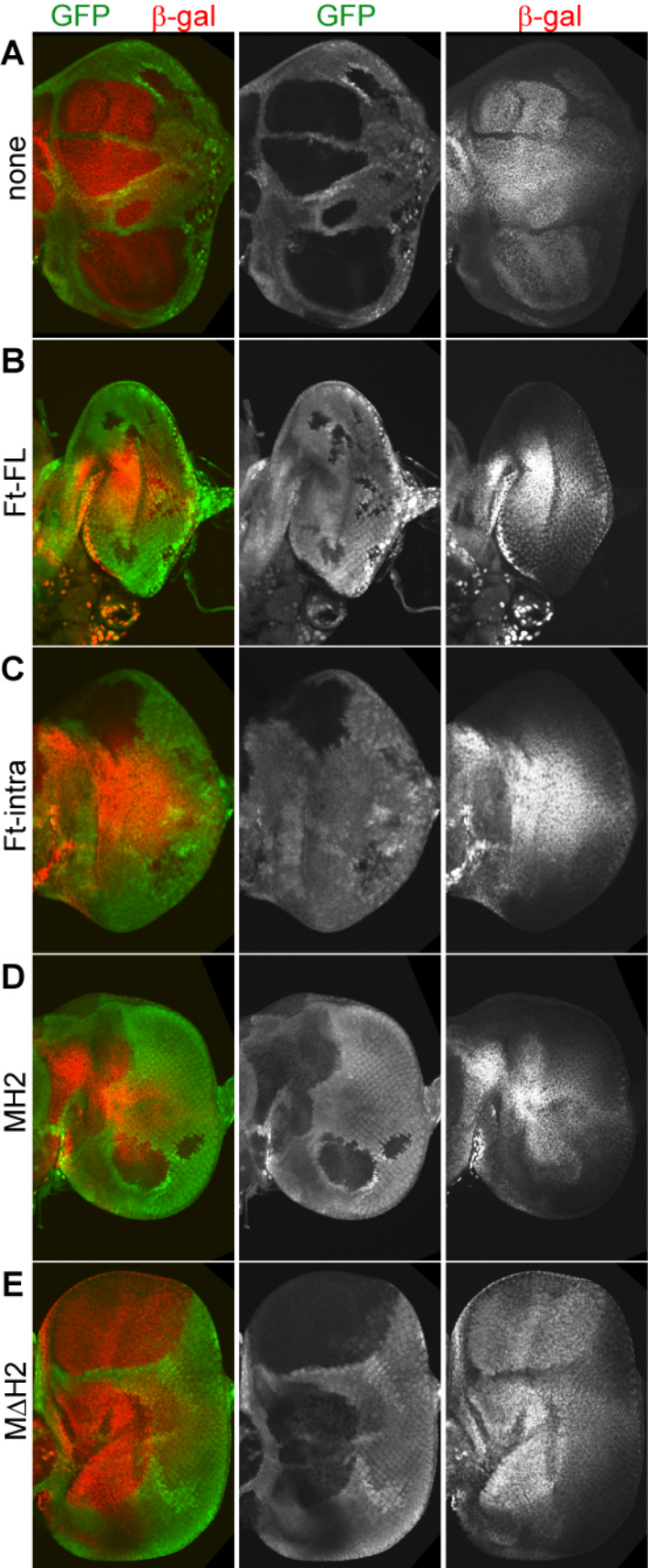

Supplement: Figure S4 — The H2 region is not sufficient to rescue elevated fj-lacZ expression in ft clones. ftG−rv mutant clones, marked by the absence of GFP expression (green channel), were generated in third-instar eye imaginal discs in which Ft mutant transgenes were expressed by TubP-Gal4. β-gal staining was shown in red. (A) ft mutant clones show autonomous increases in fj-lacZ. Ubiquitous expression of Ft-FL (B), and Ft-intra (C) rescues the elevated fj-lacZ levels in ft clones, whereas MH2 (D) and MΔH2 (E) do not rescue (see result section). (PDF) [file pone.0062998.s004.pdf]
